# Comparison of open and laparoscopic inguinal hernia repair in the elderly patients: a randomized controlled trial

**DOI:** 10.1007/s10029-025-03368-x

**Published:** 2025-05-23

**Authors:** Mehmet Esref Ulutas, Abdullah Hilmi Yilmaz

**Affiliations:** 1 Department of Surgery, University of Health Science, Gaziantep City Hospital, Şahinbey/Gaziantep, Turkey; 2 Department of Surgery, University of Health Science, Van Training and Research Hospital, Van, Turkey

**Keywords:** Complication, Elderly, Geriatric, Inguinal hernia, Laparoscopic, Open

## Abstract

**Purpose:**

It is well known that inguinal hernia repair in geriatric patients carries a higher risk of postoperative complications compared to younger patients. One of the key factors influencing these complications is the surgical technique employed. However, there is limited knowledge regarding the impact of laparoscopy on elderly patients. This prospective randomized study aims to compare the outcomes of laparoscopic and open hernia repair techniques in this patient population.

**Methods:**

Between April 2023 and April 2024, 120 consecutive patients aged 65 years and older with inguinal hernia were randomly assigned to one of two groups: the laparoscopic TEP group (*n* = 60) and the open (Lichtenstein) procedure group (*n* = 60). The study was registered at ClinicalTrials.gov (NCT06417346). The primary outcome of this study was the comparison of postoperative complication rates. Secondary outcomes included comparisons of hernia types, operative times, postoperative pain levels, and recurrence rates.

**Results:**

A total of 120 patients were followed up for 12 months. The mean age was 71.7 ± 6.5 years in the open group and 69.6 ± 3.9 years in the TEP group (*p* = 0.18). The mean operative time was 35.8 ± 7.8 min in the open group and 36.3 ± 8.7 min in the TEP group (*p* = 0.92). The mean time to return to normal daily activities was 10.6 ± 4.3 days in the open group and 7.5 ± 2.4 days in the TEP group (*p* < 0.001). On postoperative day 1, the VAS score was 3.7 ± 1.3 in the open group and 2.9 ± 1.1 in the TEP group (*p* < 0.001). At the end of the first month, the VAS score was 2.6 ± 1.0 in the open group and 1.7 ± 0.9 in the TEP group (*p* < 0.001). Chronic pain was observed in 6 patients (10%) in the open group and 1 patient (1.7%) in the TEP group (*p* = 0.05). No complications occurred in 51 patients (85%) in the open group and 52 patients (86.7%) in the TEP group (*p* = 0.84). Recurrent inguinal hernia was detected in 4 patients (6.7%) in the open group and 1 patient (1.7%) in the TEP group (*p* = 0.17).

**Conclusion:**

Based on the data obtained from our study, laparoscopic inguinal hernia repair in elderly patients was found to offer advantages such as faster recovery, reduced postoperative and chronic pain, without an increase in complications. Given these benefits, laparoscopic hernia repair can be considered a safe and preferable approach for elderly patients.

**Trial registration:**

Clinical trials number: NCT06417346.

## Introduction

The proportion of the elderly population continues to increase worldwide. Consequently, the development of specialized treatment strategies for this population has become inevitable. Inguinal hernia is one of the most common conditions in the elderly requiring surgical intervention [1]. Various open and endoscopic/laparoscopic surgical techniques have been developed for its treatment.

Globally, the use of laparoscopic surgery for inguinal hernia repair is steadily increasing, as it is in many other surgical fields. According to the HerniaSurge International Guidelines, laparo-endoscopic hernia repair techniques are more cost-effective than open hernia repair. Additionally, they offer advantages such as a lower risk of postoperative chronic pain and faster postoperative recovery.

However, laparoscopic surgery has not yet been standardized for inguinal hernia treatment due to certain disadvantages, including a long learning curve and the requirement for general anesthesia in patients [2].

Geriatric patients are known to be at a higher risk for cardiovascular, pulmonary, and urinary complications in the postoperative period following hernia repair [3]. For this reason, the impact of different surgical techniques on these complications has been a subject of ongoing interest. In particular, the effect of laparoscopic hernia repair on postoperative complications in this population is of significant importance.

The effects of laparoscopic and open inguinal hernia surgery in the elderly have been investigated and compared in several studies, albeit in limited numbers. However, these studies are predominantly observational and retrospective in nature [4–7].

Findings from review studies on this topic indicate that more randomized controlled trials are needed [8]. Therefore, this study was designed to address this gap. The aim of the study was to compare the surgical outcomes and postoperative effects of laparoscopic and open inguinal hernia repair in the elderly population (> 65 years).

## Materials and methods

### Trial design

This prospective randomized study was conducted in the General Surgery Department of the University of Health Sciences, Van Training and Research Hospital. The Ethics Committee for Clinical Studies of the University of Health Sciences, Van Training and Research Hospital, approved the study (Nr: 2023/21 − 01, Date: 04/10/23), and written informed consent was obtained from all participants. The study protocol adhered to the CONSORT guidelines and was registered at ClinicalTrials.gov (NCT06417346) [9]. All procedures involving human participants were conducted in accordance with the ethical standards of the institutional research committee, as well as the 1964 Declaration of Helsinki and its subsequent amendments or comparable ethical standards.

### Participants and eligibility criteria

The study population consisted of patients who underwent inguinal hernia repair between April 2023 and April 2024. Among these patients, those aged over 65 years constituted the study sample.

Inclusion criteria: Patients over 65 years of age, both male and female, with unilateral inguinal hernia, who underwent surgery under elective conditions.

Exclusion criteria: Patients under 65 years of age, those who underwent emergency surgery due to incarceration and/or strangulation, those with bilateral hernia, and those with contraindications to general anesthesia or spinal anesthesia.

### Sample size

The sample size was calculated based on the postoperative complication rate. Group sample sizes of 53 per group were determined to achieve 80% power to detect a 20% difference between the two groups, with a significance level of 0.05, using a two-sided Mann-Whitney U test. A total of 120 patients were enrolled to account for potential loss to follow-up.

### Randomization

All 120 included patients were randomly assigned to two groups: the Lichtenstein procedure group or the laparoscopic totally extraperitoneal (TEP) inguinal hernia repair group, using an open-label technique (1:1).

### Surgical methods

Compared to other patient groups, elderly individuals are more prone to frailty and adverse health outcomes. For this reason, in addition to a multidisciplinary preoperative evaluation aimed at the optimization of comorbidities such as diabetes mellitus, coronary artery disease, chronic kidney disease, and chronic obstructive pulmonary disease, our center implements a comprehensive prehabilitation protocol. This protocol includes individualized nutritional counseling, supervised physical exercise programs tailored to the patient’s capacity, and structured smoking cessation support. The objective is to enhance patients’ functional status prior to surgery and reduce postoperative complications.

Open (Lichtenstein) procedure: The patient was prepared under spinal anaesthesia. Following a classic inguinal incision of approximately 5–7 cm extending laterally from the pubic tubercle, the external oblique aponeurosis was opened, the external ring was disrupted, and the spermatic cord/round ligament was suspended. The hernia sac was isolated from surrounding tissues and the spermatic cord/round ligament, then either reduced or ligated. Subsequently, a polypropylene mesh measuring approximately 60 × 110 mm² was placed to completely cover the transverse fascia, and continuous sutures were used to secure it laterally along the transverse arch starting from the pubic tubercle. Hemostasis was achieved, and the layers and skin were anatomically closed.

Laparoscopic totally extraperitoneal (TEP) inguinal hernia repair: The patient was prepared under general anaesthesia. Following sterile field isolation in the supine position, an incision was made on the ipsilateral side of the inguinal hernia near the umbilicus. The incision was deepened until the anterior rectus sheath was reached. The anterior rectus sheath was cut, the rectus muscle was lateralized with a retractor, the posterior rectus sheath was visualized and the first 10 mm trocar was placed. After the 10 mm trocar insertion, insufflation was achieved with 12 mmHg pressure and telescopic bluntly dissection was begun from midline to pubic bone. In the midline, a 5 mm second trocar was entered 3–4 cm below the umbilicus and a 5 mm third trocar was entered 3–4 cm below this trocar under direct vision. Using laparoscopic dissectors and graspers, all steps of myopectineal orifice dissection were performed [10]. A 15 × 10 cm prolene mesh was spread and secured to cover both direct and indirect hernia areas, extending approximately 2–3 cm beyond. Trocars were removed under camera surveillance after CO2 desufflation, and the skin was closed.

All patients were administered parenteral paracetamole and oral acetaminophen as analgesia. Moreover, tramadol was administered intravenously to patients with VAS ≥ 4 from the 6th postoperative hour.

### Outcomes

All clinical data, including demographic characteristics such as age, sex, body mass index (BMI), comorbidities, and American Society of Anesthesiologists (ASA) scores, were recorded. The duration of the operation (skin-to-skin), types of inguinal hernias, postoperative complications, length of hospitalization, time to return to daily activities, follow-up period, and numerical pain scores on postoperative day 1, month 1, and year 1 were also recorded. Chronic pain condition and recurrence status were documented. Patients were scheduled for follow-up at the first, sixth, and twelfth months. All postoperative complications were classified according to the Clavien-Dindo classification.

Return to daily activities was defined as returning to work for employed individuals and performing all household chores without assistance. Chronic pain was defined as moderate pain that persists for at least three months.

The primary outcome of this study was postoperative complications, and the secondary outcomes were hernia recurrence, pain, and other clinical parameters.

### Follow-up period

The minimum follow-up period was 12 months. After discharge, all patients were followed up at the end of the first month, and subsequently once every three months in the outpatient clinic. Patients were instructed not to lift heavy objects for up to 2 months post-surgery. They were also advised to report to the hospital if they experienced any complaints. During follow-up visits, all patients were interviewed and examined. Patients who died or were lost to follow-up were excluded from the study. All follow-up procedures were conducted by a separate surgeon who was not involved in this study and was unaware of the randomization and patient group assignments. At the end of 12 months, a physical examination was performed on all patients. Ultrasonography, and if necessary, computed tomography, were ordered for patients with any suspicion based on physical examination. During follow-ups, all patients were asked to rate their pain level using a Visual Analog Scale (VAS) from 0 to 10.

### Statistical analyses

Following the conduction of Kolmogorov-Smirnov normality test, analyses were carried out. In case we could not provide normality within one group, we were used non-parametric test methods. Then, Mann-Whitney U test was used to compare variables between two groups.

To compare categoric variables chi square and Fisher Extract tests were used. All risk factors and possibilities regarding the analyses were presented in tables with 95% confidence intervals and p values.

All comparable results and other characteristics were given in qualitative variable rates and quantitative variables were shown in mean and median values (min-max).

For analyses, SPSS, version 22.0 (SPSS Inc., Chicago, IL, USA), software was used and *p* < 0.05 was accepted to be statistically significant value for the results.

## Results

### Demographic, clinical and surgical parameters

**Fig. 1:**
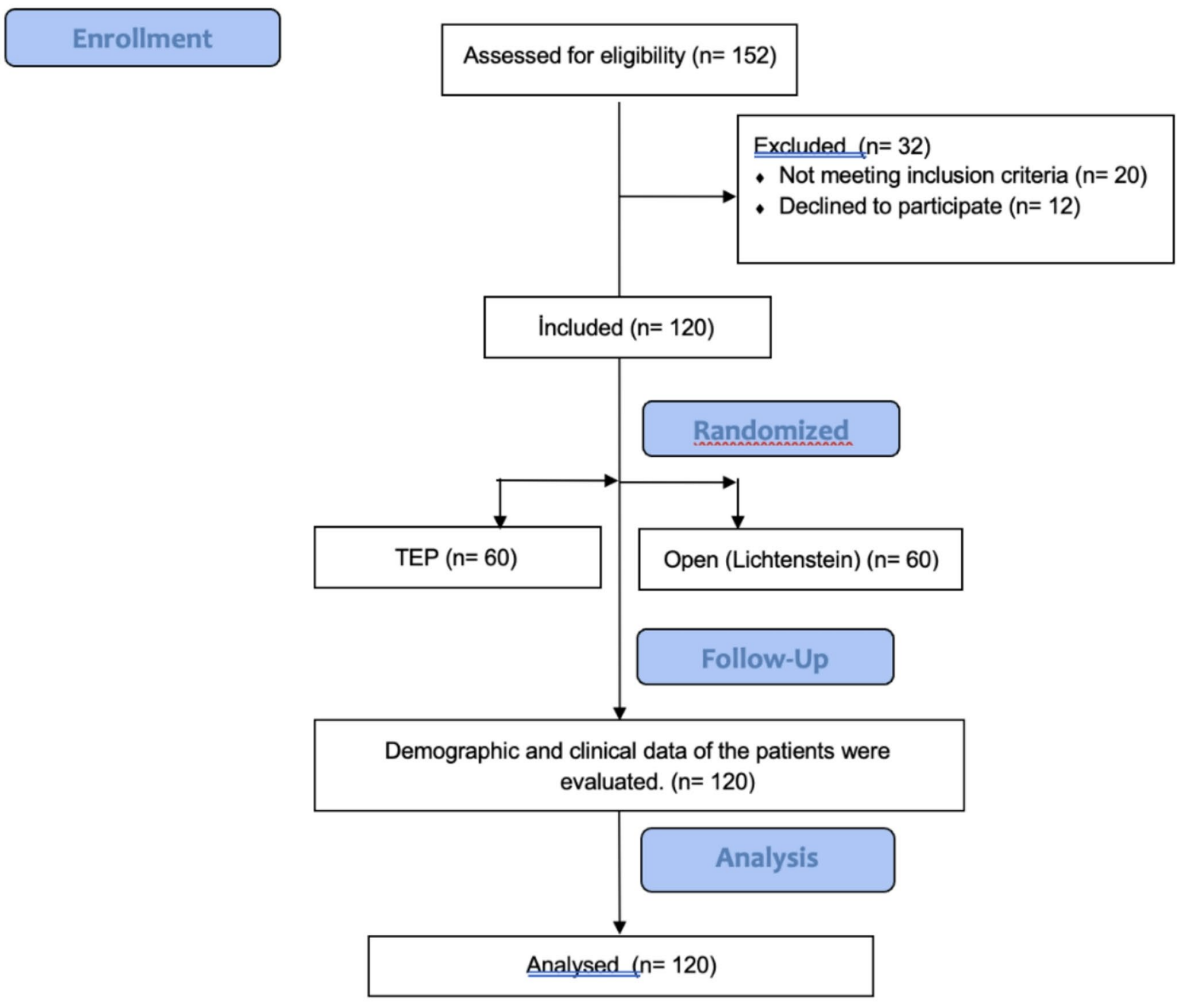
Flow Diagram

A total of 120 patients, with 60 in the open (Lichtenstein) group and 60 in the TEP group, were included in the study. In none of the cases was it necessary to switch between surgical techniques. All patients were followed up for 12 months (Fig. 1). The mean age was 71.7 ± 6.5 years in the open (Lichtenstein) group and 69.6 ± 3.9 years in the TEP group (*p* = 0.18). Forty-two patients (70%) in the open (Lichtenstein) group and 41 patients (68.3%) in the TEP group had comorbidities such as diabetes, hypertension, and coronary artery disease (*p* = 0.84). In the open (Lichtenstein) group, 21 patients (35%) were classified as ASA I, 35 patients (58.3%) as ASA II, and 4 patients (6.7%) as ASA III. In the TEP group, 4 patients (6.7%) were ASA I, 44 patients (73.3%) were ASA II, and 12 patients (20%) were ASA III (*p* < 0.001). The mean BMI of patients in the open (Lichtenstein) group was 25 ± 2.3, while that in the TEP group was 25.8 ± 2.8 (*p* = 0.07) (Table 1).

**Table 1:** Comparison of demographic characteristics of patients

	Lichtenstein (*n* = 60)	TEP (*n* = 60)	*p*
Age (year)	71.7 ± 6.5	69.6 ± 3.9	0.18
Gender (Male/Female)	60 / 0	60 / 0	-
ASA			
I	21 (% 35)	4 (% 6.7)	**< 0.001**
II	35 (% 58.3)	44 (% 73.3)	**< 0.001**
III	4 (% 6.7)	12 (% 20)	**< 0.001**
Comorbidity	42 (% 70)	41 (% 68.3)	0.84
BMI (kg/m^2^)	25 ± 2.3	25.8 ± 2.8	0.07

ASA: American Society of Anaesthesiologists, BMI: Body mass index

Patients were compared in terms of hernia classification. In the open (Lichtenstein) group, 34 patients (56.7%) had right-sided hernias and 26 patients (43.3%) had left-sided hernias. In the TEP group, 36 patients (60%) had right-sided hernias and 24 patients (40%) had left-sided hernias (*p* = 0.71). In the open (Lichtenstein) group, 37 hernias (61.7%) were indirect, 18 (30%) were direct, 2 (3.3%) were femoral, and 3 (5%) were lipomas. In the TEP group, 37 hernias (61.7%) were indirect, 17 (28.3%) were direct, 2 (3.3%) were femoral, and 4 (6.7%) were lipomas (*p* = 0.98) (Table 2).

**Table 2:** Comparison of clinical characteristics of patients

	Lichtenstein (*n* = 60)	TEP (*n* = 60)	*p*
Hernia classification			
İndirect	37 (% 61.7)	37 (% 61.7)	0.98
Direct	18 (% 30)	17 (% 28.3)	0.98
Femoral	2 (% 3.3)	2 (% 3.3)	0.98
Lipoma	3 (% 5)	4 (% 6.7)	0.98
Side of hernia			
Right	34 (% 56.7)	36 (% 60)	0.71
Left	26 (% 43.3)	24 (% 40)	0.71
Operation time (min.)	35.8 ± 7.8	36.3 ± 8.7	0.92
Length of stay (day)	1	1	-
Follow up (month)	12	12	-
Recurrence	4 (% 6.7)	1 (% 1.7)	0.17
Return to daily activities (day)	10.6 ± 4.3	7.5 ± 2.4	**< 0.001**

The mean operation time was 35.8 ± 7.8 min in the open (Lichtenstein) group, while it was 36.3 ± 8.7 min in the TEP group (*p* = 0.92). The length of hospitalization was 1 day for all patients. The mean time to return to normal daily activities was 10.6 ± 4.3 days in the open (Lichtenstein) group and 7.5 ± 2.4 days in the TEP group (*p* < 0.001) (Table 2).

### Complications

**Fig. 2:**
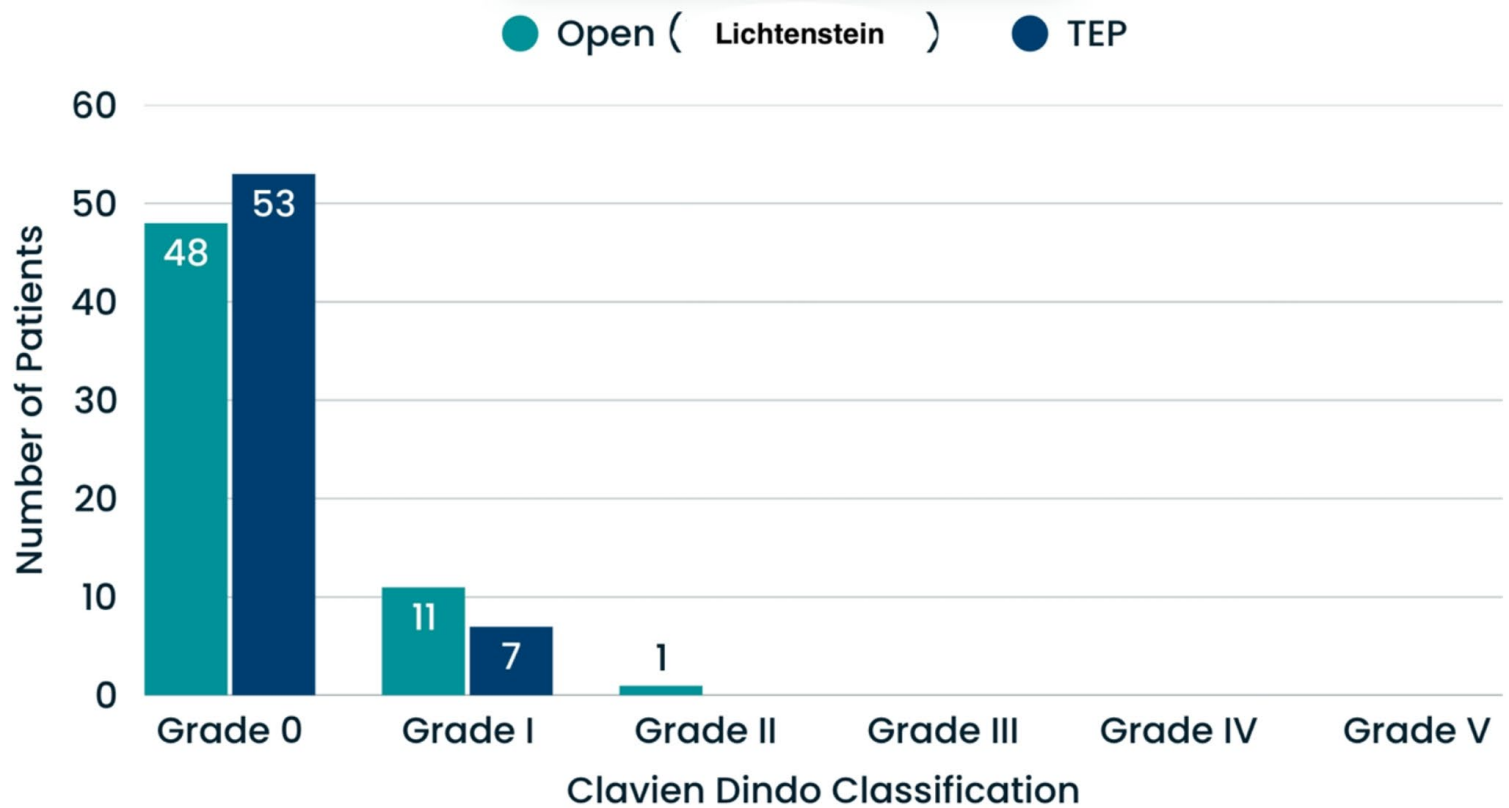
Classification of complications according to the Clavien Dindo Classification

Chronic pain was observed in 6 patients (10%) in the open (Lichtenstein) group and in 1 patient (1.7%) in the TEP group (*p* = 0.05). When comparing complications between the groups, hematoma was observed in 5 patients (8.3%) in the open (Lichtenstein) group, in 3 patients (5%) seroma, and in 1 patient (1.7%) surgical site infection. In the TEP group, hematoma was observed in 3 patients (5%), seroma in 3 patients (5%), and surgical site infection in 2 patients (3.3%). No complications were observed in 51 patients (85%) in the open (Lichtenstein) group and 52 patients (86.7%) in the TEP group (*p* = 0.84). A detailed comparison of complications according to the Clavien-Dindo classification is provided in Table 3 (Fig. 2).

**Table 3:** Comparison of patients in terms of complications and clavien Dindo classification

	Lichtenstein (*n* = 60)	TEP (*n* = 60)	*p*
Chronic Pain	6 (% 10)	1 (% 1.7)	**0.05**
Complication			
None	51 (% 85)	52 (% 86.7)	0.84
Hematoma	5 (% 8.3)	3 (% 5)	0.84
Seroma	3 (% 5)	3 (% 5)	0.84
Surgical Site İnfection	1 (% 1.7)	2 (% 3.3)	0.84
Clavien Dindo Classification			
Grade 0	48 (% 80)	53 (% 88.3)	0.34
Grade I	11 (% 18.3)	7 (% 11.7)	0.34
Grade II	1 (% 1.7)	0	-
Grade III	0	0	-
Grade IV	0	0	-
Grade V	0	0	-

### Recurrence

Recurrent inguinal hernia was observed in 4 patients (6.7%) in the open (Lichtenstein) group and in 1 patient (1.7%) in the TEP group (*p* = 0.17) (Table 2).

### VAS scores

**Fig. 3:**
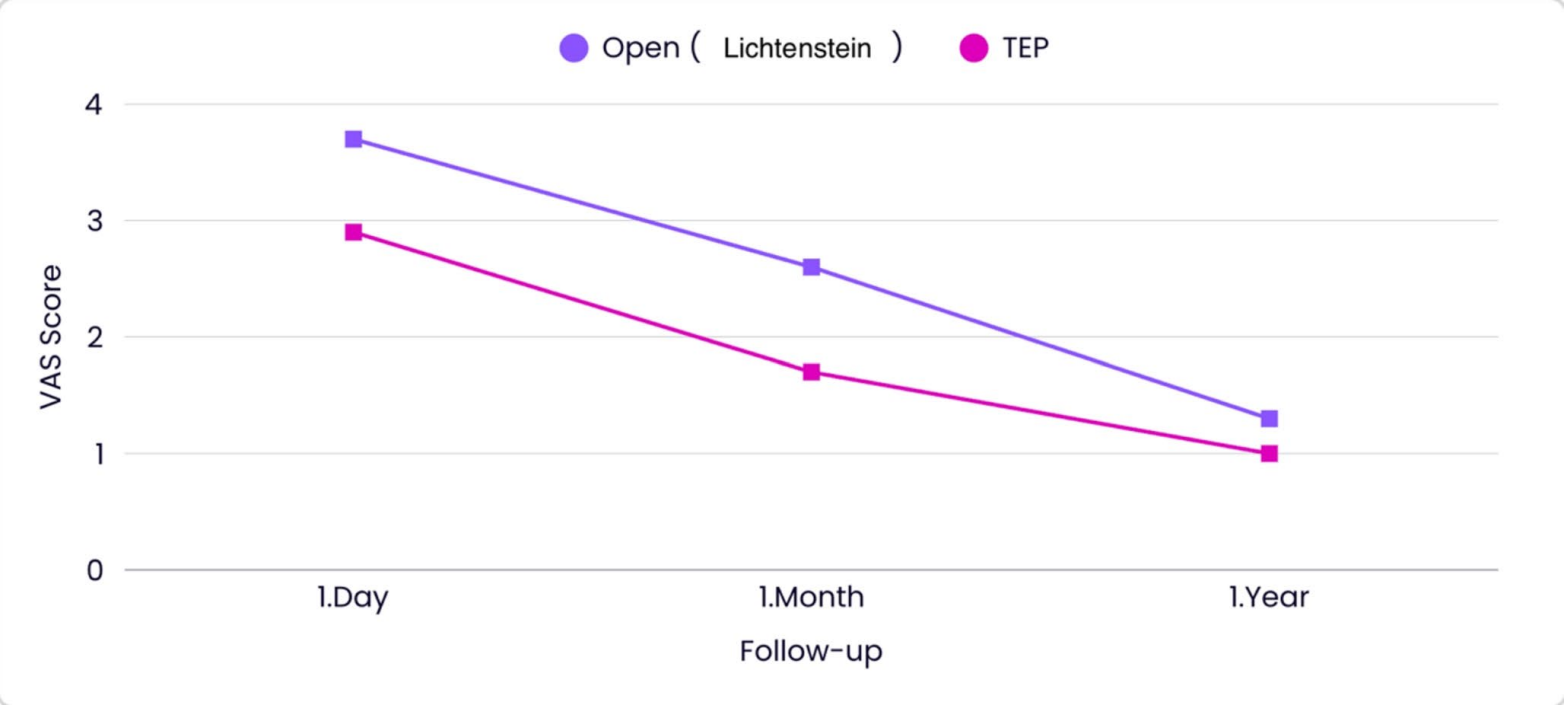
Change of VAS score over time

Patients were evaluated based on their VAS scores after surgery. On day 1, the VAS score was 3.7 ± 1.3 in the open (Lichtenstein) group and 2.9 ± 1.1 in the TEP group (*p* < 0.001). At the end of the first month, the VAS score was 2.6 ± 1 in the open (Lichtenstein) group and 1.7 ± 0.9 in the TEP group (*p* < 0.001). At the end of the first year, the VAS score was 1.3 ± 1.3 in the open (Lichtenstein) group and 1 ± 0.9 in the TEP group (*p* = 0.49) (Table 4; Fig. 3).

**Table 4:** Comparison of patients in terms of VAS scores

VAS Score	Lichtenstein (*n* = 60)	TEP (*n* = 60)	*p*
1.Day	3.7 ± 1.3	2.9 ± 1.1	**< 0.001**
1.Month	2.6 ± 1	1.7 ± 0.9	**< 0.001**
1.Year	1.3 ± 1.3	1 ± 0.9	0.49

## Discussion

There are a limited number of studies comparing open and laparoscopic procedures for the treatment of inguinal hernia, a common surgical problem in geriatric patients. Many of these studies are retrospective and observational. Systematic reviews that include these studies have highlighted the need for new prospective randomized trials [8].

In this prospective randomized study, the clinical outcomes of open (Lichtenstein) and laparoscopic inguinal hernia repair in geriatric patients were discussed. No mortality was detected in any of the 120 patients included in the study, and all patients completed their follow-ups, resulting in the completion of the study with 120 patients.

In our study, the most common type of hernia observed in patients was indirect hernias, with right-sided hernias being more frequent. Similarly, previous studies have reported that right-sided and indirect inguinal hernias are most commonly seen in the elderly population [11, 12].

When comparing the patients in terms of operative times and length of hospital stay, no significant difference was found between the laparoscopic and open (Lichtenstein) techniques. Similarly, Çiftçi and Hernandez-Rosa reached the same conclusion in their studies comparing these two techniques [13, 14].

In our study, the time to return to daily activities was significantly shorter in the laparoscopic group. This supports the findings of numerous previous studies, which have highlighted that laparoscopic inguinal hernia surgery leads to a faster recovery process [15–17]. Another important result of our study is that the method of hernia repair, whether laparoscopic or open (Lichtenstein), had no impact on recurrence rates. Similar findings have been reported in many prior studies as well [5, 15, 16, 18].

One of the most important topics investigated in this study was undoubtedly postoperative complications. In our study, no complications were detected in the majority of patients in both groups (85–86%). The complications that did develop were generally related to the surgical site, such as hematoma, seroma, and infection. These were managed with bedside interventions and medical treatments. When the complications between the laparoscopic and open (Lichtenstein) surgical groups were compared according to the Clavien-Dindo classification, no significant difference was found. A key finding of our study is that laparoscopic hernia surgery did not lead to an increase in anesthesia- or surgery-related complications in elderly patients. In a previous study investigating the feasibility of TEP repair in elderly patients, Chung et al. reported that this technique can be safely applied without differences in perioperative complications or recurrence rates [6]. In a study by Ertekin et al., laparoscopic inguinal hernia surgery was found to have lower complication rates and faster recovery times compared to open surgery, making it a safe option for elderly patients [18]. Aly et al. reported that, among surgeons, the open technique remains the most commonly used method for the elderly population, but laparoscopic surgery can be safely performed, with lower overall morbidity, surgical site infections, and reoperation risks compared to open surgery [19]. A study examining patients in their eighties and nineties found that the frequency of postoperative complications and readmission rates were significantly higher in the open hernia group compared to the laparoscopic group [20]. Hernandez-Roza et al., in their retrospective study, stated that laparoscopic inguinal hernia repair in patients in their eighties is a safe alternative with similar morbidity and mortality rates to open surgery [13].

When comparing patients in terms of pain, our study showed that, particularly on postoperative day 1 and at the 1-month mark, the VAS score in the laparoscopic group was significantly lower than in the open (Lichtenstein) surgery group. However, by the end of the 1-year follow-up, the VAS scores in both groups were very low, and there was no significant difference between the groups. This suggests that laparoscopic hernia surgery has a positive effect on pain, especially in the early postoperative period. Ertekin et al. reported that laparoscopic hernia surgery offers advantages such as lower postoperative pain scores and faster recovery times in elderly patients [18]. Similarly, Dallas et al. found that laparoscopic inguinal hernia repair provided significantly shorter pain duration and recovery time compared to open inguinal hernia repair in patients around 80 years of age, without an increase in complications [15].

In our study, chronic pain, which is a common issue in hernia surgery, was also evaluated, and it was found to be significantly lower in the laparoscopic surgery group. Some previous studies and meta-analyses have also indicated that laparoscopic hernia surgery has advantages in terms of chronic pain [21, 22]. Additionally, the use of a tacker in mesh fixation was not applied in the laparoscopic hernia repair technique (TEP) used in our study, which may have contributed to the lower incidence of chronic pain in these patients. One of the factors contributing to chronic pain is recurrence. In our study, the recurrence rate was higher in the open (Lichtenstein) repair group, which may have led to an increased number of patients with chronic pain in this group.

One of the major limitations of our study is the lack of a long-term follow-up period necessary to detect recurrence cases. However, since the study primarily focused on postoperative complications, the follow-up duration was kept short. The investigation of recurrence in these patients could be the subject of a separate study. Another limitation is that patients in the open (Lichtenstein) group underwent surgery under spinal anesthesia, while those in the laparoscopic group received general anesthesia. The differences in anesthesia techniques between the groups may have affected the evaluated parameters. Studies comparing these surgical methods under the same anesthesia protocols could provide more reliable results.

Another limitation of our study is related to its design, as the group allocation of patients was not known at the beginning due to the randomized nature of the trial. The type of anesthesia to be administered was determined based on the assigned treatment group. Therefore, patients with contraindications to either general or spinal anesthesia were excluded at the outset. This exclusion was necessary to maintain proper randomization. However, omitting this subset of patients introduced a limitation regarding the generalizability of the findings, particularly in terms of representing the entire elderly patient population.

Given the increasing number of elderly patients undergoing hernia repair, future studies could focus on comparing open hernia repair performed under spinal anesthesia with alternative, less invasive anesthetic techniques. In particular, the use of local anesthesia or truncal blocks with targeted ultrasound-guided infiltration—considered to be lighter and potentially safer options in patients of advanced age—may offer valuable insights into optimizing perioperative management in this population.

## Conclusion

Considering the data obtained from our study and similar studies in the literature, it has been determined that laparoscopic inguinal hernia repair in elderly patients offers advantages such as faster recovery, less postoperative pain, and a lower incidence of chronic pain without causing an increase in complications. Due to these advantages, laparoscopic hernia repair can be safely considered a preferable method in elderly patients.
