# Correction to: Comparison of open and laparoscopic inguinal hernia repair in the elderly patients: a randomized controlled trial

**DOI:** 10.1007/s10029-025-03514-5

**Published:** 2025-11-10

**Authors:** Mehmet Esref Ulutas, Abdullah Hilmi Yilmaz

**Affiliations:** 1 Department of Surgery, University of Health Science, Gaziantep City Hospital, Şahinbey/Gaziantep, Turkey; 2 Department of Surgery, University of Health Science, Van Training and Research Hospital, Van, Turkey

**Correction to: Hernia _#####################_**

10.1007/s10029-025-03368-x

The dates in the Methods of the Abstract and Participants and Eligibility Criteria sections tions have been published incorrectly.

1. Abstract Methods Section: **Between April 2023 and April 2024, 120 consecutive patients aged 65 years and older with inguinal hernia were randomly assigned to one of two groups: the laparoscopic TEP group (n=60) and the open (Lichtenstein) procedure group (n=60).]**
  Should read
  Abstract Methods Section: **Between October 2023 and December 2024, 120 consecutive patients aged 65 years and older with inguinal hernia were randomly assigned to one of two groups: the laparoscopic TEP group (n=60) and the open (Lichtenstein) procedure group (n=60).]**
2. Participants and eligibility criteria section: The study population consisted of patients who underwent inguinal hernia repair between April 2023 and April 2024.
  Should read
  Participants and eligibility criteria section: **The study population consisted of patients who underwent inguinal hernia repair between October 2023 and December 2024.]**

